# Postoperative cervical length to predict success of repeat cerclage in singleton pregnancies with prolapsed membranes after prior cerclage

**DOI:** 10.3389/fmed.2023.1248321

**Published:** 2023-08-21

**Authors:** Suyeon Park, Keun-Young Lee, Ji-Eun Song

**Affiliations:** ^1^Department of Obstetrics and Gynecology, Inha University College of Medicine, Inha University Hospital, Incheon, Republic of Korea; ^2^Department of Obstetrics and Gynecology, Hallym University College of Medicine, Kangnam Sacred Heart Hospital, Seoul, Republic of Korea

**Keywords:** repeat cerclage, singleton pregnancy, preterm birth, cervical length, prior cerclage, membrane bulging

## Abstract

**Background:**

This study aimed to evaluate the outcome of repeat cerclage (RC) in singleton pregnancies with prolapsed membranes following a prior cerclage and analyze predictive factors for delivery at ≥26 weeks of gestation following RC.

**Materials and methods:**

Patients who underwent RC between 2010 and 2020 at the Hallym University Medical Center were reviewed. Women with singleton pregnancies with prolapsed membranes following prior cerclage were candidates for RC. We analyzed the characteristics, pregnancy outcomes, perioperative clinical and laboratory findings, and postoperative cervical length (CL) to identify the factors for predicting delivery at ≥26 weeks following RC.

**Results:**

Thirty-five women with RC were identified; the median gestational age (GA) at a prior cerclage was 14 weeks, the average GA at RC was 21 + 3 weeks, and the median GA at delivery following RC was 26 + 2 weeks. Patients were divided into two groups based on their delivery status at 26 weeks: 17 women delivered at <26 weeks (range, 18 + 4–25 + 6 weeks) (Group A) and 18 women delivered at ≥26 weeks (range, 26 + 2–40 + 3 weeks) (Group B). The median GA at delivery in group A was 22 + 4 weeks, whereas that in group B was 33 + 4 weeks (*p* < 0.001). No differences in preoperative clinical and laboratory findings were observed between the two groups. However, the postoperative CL in group A was significantly shorter than that in group B (12 mm vs. 21.5 mm, *p* < 0.001). The ROC curve of postoperative CL predicting delivery at ≥26 weeks showed an AUC of 0.843; a CL of 20 mm showed a sensitivity of 61.1% and a specificity of 100%.

**Conclusion:**

RC may prolong singleton pregnancies with prolapsed membranes following prior cerclage. A postoperative CL ≥20 mm may predict the success of RC.

## Background

Cervical insufficiency, defined as painless cervical dilatation in the mid-trimester, is a well-known cause of preterm delivery, accounting for 10–25% of all second-trimester pregnancy losses (1). Singleton pregnancies with bulging membranes are associated with a poor prognosis, and cerclage indicated by physical examination is known to be an effective procedure for reducing preterm birth, neonatal morbidity, and mortality (2).

Membrane bulging even after prior cerclage is considered a failure of the cerclage. When this happens, many patients do not opt to pursue further intervention and choose to untie the prior cerclage and deliver soon. No absolute guidelines exist for the treatment of bulging membranes following prior cerclage. Prior studies suggest repeat cerclage (RC) to treat women with bulging membranes, even after a prior cerclage. Our previous study reported that RC could prolong pregnancy compared to bed rest (3). However, limited data are available on the efficacy of RC and predictive factors for its success. The aim of our study was to evaluate the outcomes of RC in singleton pregnancies with prolapsed membranes following prior cerclage and to analyze the predictive factors for delivery at ≥26 weeks following RC.

## Materials and methods

This retrospective cohort study focused on singleton pregnancies in women who underwent RC with prolapsed membranes following a prior cerclage at the Kangnam Sacred Heart Hospital, Seoul, Korea, between January 2010 and December 2020. The study protocol was approved by the Institutional Review Board of the Kangnam Sacred Heart Hospital. The requirement for informed consent was waived owing to its retrospective nature.

We included singleton pregnancies that presented between 16 0/7 and 23 6/7 weeks of gestation, exhibiting painless bulging membranes upon speculum examination after a prior cerclage. These patients underwent RC and were subsequently delivered at the same hospital. Meanwhile, we excluded patients with twin pregnancies, chorioamnionitis, preterm premature rupture of membranes or persistent contractions, fetal anomalies, and medically-indicated preterm delivery (severe preeclampsia, fetal growth restriction, placental abruption, or placenta previa). Clinical chorioamnionitis was diagnosed based on one or more of the following criteria: maternal fever ≥38°C, maternal or fetal tachycardia, or maternal blood cell count ≥ 15,000/mm^3^.

Under general anesthesia, two physicians performed RC in the Trendelenburg position. We did not disinfect the vaginal cavity due to the risk of intraoperative membrane rupture and inserted the Foley catheter to empty the bladder. We used two Simpson retractors to expose the membranes and cervix and two atraumatic forceps to fix the cervical edges. Then, McDonald sutures were performed using a purse-string suture and 5-mm polyester tape (Cervix set, *B Braun*) after pushing the bulging membranes using a uniconcave balloon device into the uterine cavity when appropriate. Some patients who were at risk of intraoperative membrane rupture underwent amniocentesis to reduce the pressure or amount of amniotic fluid. In addition, the previous suture was maintained or removed depending on the feasibility of RC. After surgery, all patients were instructed to observe bed rest and were administered prophylactic tocolytics and cephalosporin, metronidazole, and azithromycin intravenously for at least 7 days (4).

The medical data collected for this study included maternal age, parity, rate of assisted reproductive technology (ART), prior cervical operation, and prior preterm birth history; body mass index (BMI); number of cerclage knots; laboratory findings (neutrophil count/lymphocyte count ratio, CRP before and after prior cerclage); rate of positive vaginal culture; gestational age (GA) at prior cerclage, RC, and delivery; interval from prior cerclage to RC and RC to delivery; rate of preterm birth before 26 weeks; and post-operative cervical length following RC. Other data included birth weight; the rates of very low birth weight and extremely low birth weight; an Apgar score of <7 at 5 min; the rate of neonatal mortality (viability, perinatal death before 72 weeks, and early neonatal death before 7 days); and neonatal morbidities (respiratory distress syndrome [RDS], bronchopulmonary dysplasia [BPD], necrotizing enterocolitis, intraventricular hemorrhage [IVH] grade 3 or 4, retinopathy of prematurity [ROP] requiring three or more ophthalmology office visits, and sepsis).

We evaluated the outcomes of RC in singleton pregnancies with prolapsed membranes following prior cerclage. Furthermore, we analyzed the predictive factors contributing to preterm birth before 26 weeks. Moreover, we conducted a subgroup analysis based on the postoperative CL cutoffs to investigate the efficacy of RC at certain CLs.

Descriptive statistics were calculated, with continuous data presented as medians (ranges) and categorical variables presented as numbers (percentages). Non-parametric tests were used to compare data due to the sample size (*n* = 35), and comparisons were performed using the Fisher's exact test or Mann–Whitney U-test. A multivariable logistic regression was performed to determine the variables associated with the outcomes. A receiver-operating characteristic (ROC) curve was used to obtain the cutoff value for cervical length. The area under the curve (AUC) was used as an indicator of accurate prediction. The cutoff value of each parameter was determined according to sensitivity and specificity. The data were assessed using R version 4.2.2. A *P*-value of < 0.05 was considered statistically significant.

## Results

We identified 35 women with singleton pregnancies and prolapsed membranes after a prior cerclage. Table 1 summarizes the maternal characteristics and the pregnancy and neonatal outcomes. The median GA at the time of a prior cerclage was 14 weeks (range, 12 + 5–20 + 1 weeks). Among the 35 patients, 20 women (57.1%) underwent history-indicated cerclage, 5 (14.3%) underwent ultrasound-indicated cerclage, and 10 (28.6%) underwent physical examination-indicated cerclage (PEIC). The rate of vaginal culture positivity was 55.9% (19/34), and the median cervical length following RC was 17 mm (range, 5–41 mm). In all 35 cases, surgery for RC was performed successfully without any incident of intraoperative membrane rupture or immediate pregnancy loss. However, following RC, six patients experienced premature rupture of membranes within a week (range, 2–7 days), and only one patient was diagnosed with chorioamnionitis. The median GA at RC was 21 + 3 weeks (range, 16 + 2–23 + 6 weeks). The median size of bulging membranes following the failure of a prior cerclage was 2.5 cm (range, 1.0–6.0 cm), and the rate of single and double knots was 60% (21/35) and 40% (14/35), respectively. The median GA at delivery following RC was 26+2 weeks (range, 18 + 4–40 + 3 weeks), and the median prolongation duration between RC and delivery was 32 days (range, 2–144 days). Of the 35 patients, 12 (34.3%) underwent cesarean section due to a prior cesarean section history, fetal malposition, or placenta previa, whereas 23 patients (65.7%) underwent vaginal delivery after the removal of the RC knots. The median neonatal birth weight was 1,050 g (range, 160–3,710 g); the rates of perinatal death (< 72 h) and early neonatal death (<7 days) were 14.8% (4/27) and 18.5% (5/27), respectively; eight patients (22.9%) did not achieve viability due to extreme prematurity (Table 1).

**Table 1:** Characteristics and outcomes of repeat cerclage (*n* = 35).

	**Population (*n* = 35)**
Maternal age (years)	34 [29–42]
BMI	25.2 [19.5–38.0]
**Parity**	
Primiparous	13/35 (67.1%)
Multiparous	22/35 (62.9%)
History of PTB	27/35 (77.1%)
History of conization/LEEP	1/35 (2.9%)
Mullerian anomaly	2/35 (5.7%)
Chronic comorbidities	1/35 (2.9%)
In vitro fertilization	8/35 (22.9%)
**Types of prior cerclage**	
History-indicated cerclage	20/35 (57.1%)
Ultrasound-indicated cerclage	5/35 (14.3%)
Physical examination-indicated cerclage	10/35 (28.6%)
GA at prior cerclage (weeks)	14 + 0 [12 + 5–20+1]
GA at repeat cerclage (weeks)	21 + 3 [16 + 2–23+6]
Latency between RC and prior cerclage (days)	36 [5–76]
Size of bulging membranes (cm)	2.5 [1.0–6.0]
Culture positive	19/34 (55.9%)
NLR before RC	4.9 [2.8–18.0]
CRP before RC	5.8 [0.9–44.0]
NLR after RC	6.8 [1.9–69.5]
CRP after RC	15.8 [1.8–66.4]
**Number of suture knots**	
Single knot	21/35 (60%)
Double knots	14/35 (40%)
Postoperative CL (mm)	17 [5–41]
PPROM within a week	6/35 (17.1%)
Chorioamnionitis	1/35 (2.9%)
GA at delivery (weeks)	26+2 [18 + 4–40 + 3]
Prolongation date (days)	32 [2–144]
**Mode of delivery**	
Cesarean section	12/35 (34.3%)
Vaginal delivery	23/35 (65.7%)
Neonatal birth weight (g)	1050 [160–3710]
Very low birth weight	21/35 (60%)
Extremely very low birth weight	17/35 (48.6%)
Viability	27/35 (77.1%)
NICU admission	21/27 (77.8%)
Agar score <7 at 5 min	13/27 (48.1%)
	**Population (*****n*** = **35)**
Perinatal death (<72 h)	4/27 (14.8%)
Early neonatal death (<7 days)	5/27 (18.5%)

Data are expressed as medians (ranges), and numbers (percentages).

BMI, body mass index; PTB, preterm birth; LEEP, loop electrosurgical excision procedure; GA, gestational age; RC, repeat cerclage; NLR, neutrophil lymphocyte ratio; CRP, c-reactive protein; CL, cervical length; PPROM, preterm premature rupture of membranes; NICU, neonatal intensive care unit.

Patients were divided into the following two groups based on their delivery status at 26 weeks, which was the median GA at delivery: 17 women delivered at <26 weeks (range, 18 + 4–25 + 6 weeks) (Group A) and 18 women delivered at <26 weeks (range, 26 + 2–40 + 3 weeks) (Group B) (Table 2). The median GA at delivery in group A was 22 + 4 weeks, whereas that in group B was 33+4 weeks (*p* < 0.001) (Table 3). No differences were observed in terms of preoperative clinical (BMI, parity, history of preterm birth and conization or LEEP, the rate of ART, Mullerian anomaly, vaginal culture positive, size of the bulging membrane at RC, use of amniocentesis, number of knots, and GA at RC) or laboratory findings (neutrophil–lymphocyte ratio, CRP before and after RC) between the two groups. However, the postoperative CL following RC was significantly shorter in group A than in group B (13 mm vs. 22 mm, *p* < 0.001). As expected, neonatal outcomes, such as neonatal birth weight, the incidence of very low birth weight infants and extremely low birth weight infants, an Apgar score <7 at 5 min, neonatal mortality, and morbidity were worse in group A than in group B (Table 3).

**Table 2:** Comparison of maternal characteristics between GA at delivery <26 weeks and ≥26 weeks.

	**GA at delivery <26 weeks (*n* = 17)**	**GA at delivery ≥26 weeks (*n* = 18)**	***p*-value**
Maternal age	33 [29–40]	36 [29–42]	0.02^*^
BMI	25.3 [19.5–38.0]	24.9 [22.5–28.6]	0.72
**Parity**			
Primiparous	11/17 (64.7%)	11/18 (61.1%)	1
Multiparous	6/17 (35.3%)	7/18 (38.9%)		
History of PTB	12/17 (70.6%)	15/18 (83.3%)	0.44
History of conization/LEEP	0/17 (0%)	1/18 (94.4%)	1
Mullerian anomaly	2/17 (11.8%)	0/18 (0%)	0.23
In vitro fertilization	3/17 (17.7%)	5/18 (27.8%)	0.69
**Prior cerclage type**			
History-indicated cerclage	7/17 (41.2%)	13/18 (72.2%)	0.15
Ultrasound-indicated cerclage	4/17 (23.5%)	1/18 (5.6%)		
Physical examination-indicated cerclage	6/17 (35.3%)	4/18 (22.2%)		
GA at prior cerclage (weeks)	15 [13–20+1]	13+6 [12+5–18]	0.06
GA at repeat cerclage (weeks)	21 [16+2–23+4]	21+6 [16+2–23+6]	0.24
Latency between RC and prior cerclage	30 [8–66]	49 [5–76]	0.008^*^
Size of bulging membranes (cm)	2.7 [1.0–6.0]	2.1 [1.0–5.0]	0.12
Culture positive	10/17 (58.82%)	10/18 (55.56%)	1
NLR before RC	5.2 [2.8–12.3]	4.9 [3.0–18.0]	0.55
CRP before RC	5.8 [0.9–42.3]	6.0 [3.0–18.0]	0.44
NLR after RC	7.0 [2.9–12.7]	6.5 [1.9–69.5]	0.87
CRP after RC	11.2 [1.8–54.9]	19.2 [5.1–66.4]	0.21
Use of amniocentesis	6/17 (35.3%)	12/18 (66.7%)	0.094
Number of suture knots	13/17 (76.5%)	8/18 (44.4%)	0.11
Postoperative CL (mm)	13 [8–20]	22 [5–41]	< 0.001^*^

Data are expressed as medians (ranges), and numbers (percentages).

^*^p < 0.05, which means statistical difference.

BMI, body mass index; GA, gestational age; RC, repeat cerclage; NLR, neutrophil lymphocyte ratio; CL, cervical length; PTB, preterm birth; CRP, c-reactive protein; LEEP, loop electrosurgical excision procedure.

**Table 3:** Comparison of pregnancy and neonatal outcomes between GA at delivery <26 weeks and ≥26 weeks.

	**GA at delivery <26 weeks (*n* = 17)**	**GA at delivery ≥26 weeks (*n* = 18)**	***p*-value**
GA at delivery (weeks)	22+4 [18+4–25+6]	33+4 [26+2–40+3]	< 0.001^*^
Prolongation date (days)	13 [2–52]	94 [26–144]	< 0.001^*^
PPROM within a week	6/17 (35.3%)	0/18 (0%)	0.008^*^
Chorioamnionitis	1/17 (5.9%)	0/18 (0%)	0.49
Neonatal birth weight (g)	585 [160–1,090]	2,213 [920–3,710]	< 0.001^*^
Very low birth weight	17/17 (100%)	4/18 (22.2%)	< 0.001^*^
Extremely low birth weight	16/17 (94.1%)	1/18 (5.6%)	< 0.001^*^
Viability	9/17 (52.9%)	18/18 (100%)	0.001^*^
Apgar score < 7 at 5 min	1/9 (11.1%)	13/18 (72.2%)	0.004^*^
Perinatal death (<72 h)	4/9 (44.4%)	0/18 (0%)	0.007^*^
Early neonatal death (<7 days)	5/9 (55.6%)	0/18 (0%)	0.002^*^
NICU admission	9/9 (100%)	12/18 (66.7%)	0.071
Respiratory distress syndrome	9/9 (100%)	3/18 (16.7%)	< 0.001^*^
Bronchopulmonary dysplasia	8/9 (88.9%)	3/18 (16.7%)	0.001^*^
Necrotizing enterocolitis	2/9 (22.2%)	0/18 (0%)	0.10
Intraventricular hemorrhage	3/9 (33.3%)	1/18 (5.6%)	0.09
Retinopathy of prematurity	3/9 (33.3%)	6/18 (33.3%)	1.00
Sepsis	8/9 (88.9%)	4/18 (22.2%)	0.003^*^

Data are expressed as medians (ranges), and numbers (percentages).

^*^p < 0.05, which means statistical difference.

GA, gestational age; PPROM, preterm premature rupture of membranes; NICU, neonatal intensive care unit.

**Figure 1:**
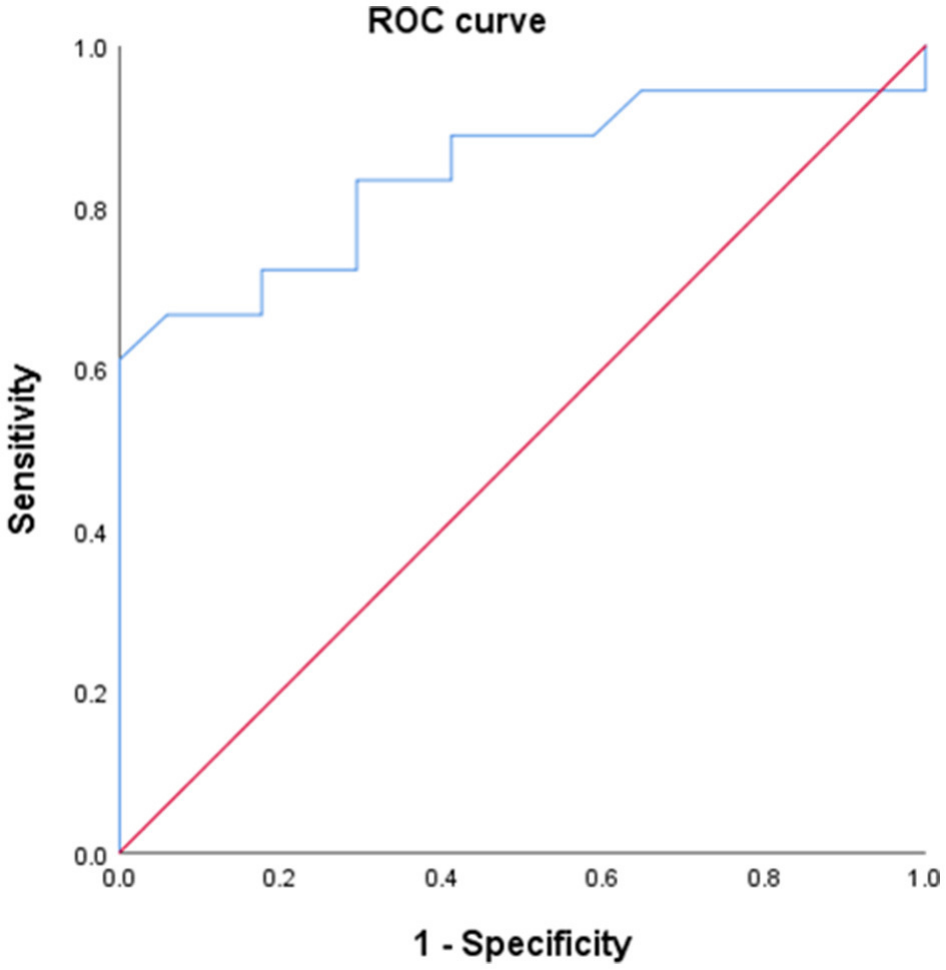
ROC curve of post-operative CL predicting delivery at ≥26 week of gestation.

To evaluate the predictive factors for preterm delivery before 26 weeks, we performed a ROC curve analysis. The ROC curve for postoperative CL following RC is shown in Figure 1. The AUC was >0.6 (0.843), and the optimal cutoff value of postoperative CL was 20 mm (sensitivity, 61.1%; specificity, 68.6%). Furthermore, we compared the pregnancy and neonatal outcomes according to postoperative CL <20 mm and ≥20 mm, which is the optimal cutoff value associated with preterm delivery before 26 weeks, to re-verify the predictive factors. In total, 22 cases of postoperative CL following RC were measured under 20 mm, whereas 13 cases were measured over 20 mm (Table 4). The postoperative CL ≥20 mm group had more history-indicated cerclages (11/13 [84.6%] vs. 9/22 [40.9%], *p* = 0.038) and underwent earlier prior cerclages (13 + 5 weeks vs. 14 + 6 weeks, *p* = 0.045) than the postoperative CL <20 mm group. No differences were observed in preoperative clinical and laboratory findings between the two groups. However, the postoperative CL <20 mm group delivered earlier (median, GA 27 + 3 weeks vs. GA 35 + 5 weeks, *p* < 0.001) and experienced a higher incidence of premature rupture of membranes within one week (27.3% vs. 0%, *p* = 0.046) than the postoperative CL ≥ 20 mm group (Table 5). The median neonatal birth weight in the postoperative CL <20 mm group was lower than that in the postoperative CL ≥ 20 mm group (665 g vs. 2,490 g, *p* < 0.001), and the incidence of very low birth weight and extremely low birth weight was also higher in the postoperative CL <20 mm group than in the postoperative CL ≥ 20 mm group. Additionally, the rates of Apgar score <7 at 5 min, NICU admission, and neonatal morbidities including RDS, BPD, and sepsis were higher in the postoperative CL <20 mm group than in the postoperative CL ≥ 20 mm group.

**Table 4:** Comparison of maternal characteristics between postoperative CL of <20 mm and ≥20 mm.

	**Postoperative CL of <20 mm (*n* = 22)**	**Postoperative CL of ≥20 mm (*n* = 13)**	***p*-value**
Maternal age	33 [29–41]	36 [29–42]	0.27
BMI	25.3 [19.5–38.0]	25.2 [20.4–28.6]	0.48
**Parity**			
Primiparous	8/22 (36.4%)	5/13 (38.5%)	1.00
Multiparous	14/22 (63.6%)	8/13 (61.5%)		
History of PTB	16/22 (72.7%)	11/13 (84.6%)	0.68
History of conization/LEEP	0/22 (0%)	1/13 (7.7%)	0.37
Mullerian anomaly	2/22 (9.1%)	0/13 (0%)	0.52
In vitro fertilization	4/22 (18.2%)	4/13 (30.8%)	0.43
**Prior cerclage type**			
History-indicated cerclage	9/22 (40.9%)	11/13 (84.6%)	0.04^*^
Ultrasound-indicated cerclage	4/22 (18.2%)	1/13 (7.7%)		
Physical examination-indicated cerclage	9/22 (40.9%)	1/13 (7.7%)		
GA at prior cerclage (weeks)	14+6 [13–20+1]	13+5 [12+5–17+4]	0.05
GA at repeat cerclage (weeks)	21 [16+2–23+4]	22+2 [16+2–23+6]	0.23
Latency between RC and prior cerclage (days)	30 [5–70]	56 [20–76]	0.02^*^
Size of bulging membranes (cm)	2.8 [1.0–6.0]	1.9 [1.0–4.0]	0.09
Culture positive	12/22 (54.5%)	7/13 (53.8%)	1.00
NLR before RC	5.0 [2.8–12.5]	4.9 [3.0–18.0]	0.36
CRP before RC	4.9 [0.9–38.3]	6.2 [2.9–44.0]	0.17
NLR after RC	6.5 [2.9–69.5]	6.8 [1.9–18.4]	0.72
CRP after RC	10.3 [1.8–54.9]	21.6 [6.0–66.4]	0.08
**Number of suture knots**			
Single knot	15/22 (68.2%)	6/13 (46.2%)	0.29
Double knots	7/22 (31.8%)	7/13 (53.8%)		
Postoperative CL (mm)	13 [5–19]	26 [20–41]	< 0.001^*^

Data are expressed as medians (ranges), and numbers (percentages).

^*^p < 0.05, which means statistical difference.

BMI, body mass index; GA, gestational age; RC, repeat cerclage; NLR, neutrophil lymphocyte ratio; CL, cervical length; PTB, preterm birth; CRP, c-reactive protein; LEEP, loop electrosurgical excision procedure.

**Table 5:** Comparison of pregnancy and neonatal outcomes between postoperative CL of <20 mm and ≥20 mm.

	**Postoperative CL at <20 mm (*n* = 22)**	**Postoperative CL at ≥20 mm (*n* = 13)**	***p*-value**
GA at delivery (weeks)	27+3 [18+4–40+1]	35+5 [24+5–40+3]	< 0.001^*^
Prolongation date (days)	22 [2–131]	96 [13–144]	< 0.001^*^
PPROM within a week	6/22 (27.3%)	0/13 (0%)	0.04^*^
Chorioamnionitis	1/22 (4.5%)	0/13 (0%)	0.63
Neonatal birth weight (g)	665 [160–2860]	2490 [830–3710]	< 0.001^*^
Very low birth weight	18/22 (81.8%)	3/13 (23.1%)	0.001^*^
Extremely low birth weight	15/22 (68.2%)	2/13 (15.4%)	0.005^*^
Viability	14/22 (51.9%)	13/13 (100%)	0.02^*^
Apgar score < 7 at 5 min	10/14 (71.4%)	3/13 (23.1%)	0.02^*^
Perinatal death (<72 h)	3/14 (21.4%)	1/13 (7.7%)	0.60
Early neonatal death (<7 days)	4/14 (28.6%)	1/13 (7.7%)	0.33
NICU admission	14/14 (100%)	7/13 (53.8%)	0.006^*^
Respiratory distress syndrome	10/14 (71.4%)	2/13 (15.4%)	0.001^*^
Bronchopulmonary dysplasia	9/14 (64.3%)	2/13 (15.4%)	0.03^*^
Necrotizing enterocolitis	2/14 (14.3%)	0/13 (0%)	0.48
Intraventricular hemorrhage	4/14 (28.6%)	0/13 (0%)	0.09
Retinopathy of prematurity	6/14 (42.9%)	3/13 (23.1%)	0.42
Sepsis	9/14 (64.3%)	3/13 (23.1%)	0.04^*^

Data are expressed as medians (ranges), and numbers (percentages).

^*^p < 0.05, which means statistical difference.

GA, gestational age; PPROM; preterm premature rupture of membranes; NICU, neonatal intensive care unit.

Furthermore, we performed a sub-analysis according to the types of prior cerclage and number of knots at RC (Supplementary Tables 1, 2). No significant differences were observed in the characteristics, pregnancy, or neonatal outcomes according to the types of prior cerclage and the number of knots at RC.

## Discussion

Our study demonstrated that RC could prolong singleton pregnancies with prolapsed membranes following a prior cerclage and decrease the incidence of preterm birth. In our study, the median GA at RC and delivery were 21 + 3 weeks (range, 16 + 2–23 + 6 weeks) and 26 + 2 weeks (range, 18 + 4–40 + 3 weeks), respectively, and RC prolonged the pregnancy date by 32 days. This indicated that patients who underwent RC following a prior cerclage achieved viability through RC. Moreover, we identified postoperative CL as a predictive factor for preterm birth before a GA of 26 weeks, as determined by various statistical methods.

Many previous studies demonstrated that emergency cerclage in pregnant women with painless cervical dilatation is beneficial in reducing preterm birth and prolonging pregnancy (5–7). A recent meta-analysis reported that emergency cerclage decreased preterm birth, and prolonged pregnancy, and decreased neonatal deaths and fetal losses without increasing the risk of chorioamnionitis or premature rupture of membranes (7). Although RC and emergency cerclage have in common that both were performed under the same condition of bulging membranes, RC has higher surgical risks than emergency cerclage. Unfortunately, there are fewer studies on the efficacy of RC compared to emergency cerclage.

The effect of RC has been a subject of controversy. Baxter et al. reported that, compared to expectant management, RC in the short cervix was associated with earlier delivery (8). Contag et al. also demonstrated that RC in the short cervix after a prior cerclage did not prolong the duration of pregnancy (9). However, these studies only included cases of short cervix identified through ultrasonography and did not focus on cases with a dilated cervix or bulging membranes. Perhaps these differences in the sample population may have caused inconsistent outcomes.

Cai et al. analyzed the effect of RC on prolapsed membranes and observed that RC delayed delivery by an additional 5 weeks (10). Furthermore, in a previous study, we evaluated 22 patients with bulging membranes following a prior cerclage, 11 of whom underwent RC and the other 11 received conservative treatment. The RC group had a significantly longer prolongation of pregnancy (35.8 days) than that of the non-RC group (1.4 days) (3). Based on the previous study, we recommended RC in women with prolapsed membranes after prior cerclage and collected additional data from 35 women with RC in singleton pregnancies. Our current study also demonstrated a consistent prolongation of pregnancy following RC (32 days), which aligns with the findings of our previous study.

Although few previous studies have reported on the simple effects of RC, such as pregnancy prolongation (3–5), limited data are available to identify the predictive factors, as in our study. We conducted a thorough analysis of various factors using multiple statistical trials to identify the predictive factors for the prevention of preterm birth and demonstrated an association between postoperative CL following an RC and preterm delivery before 26 weeks of gestation. Notably, as postoperative CL increased, the risk of preterm birth before 26 weeks decreased. Postoperative CL > 20 mm following RC may be a predictive factor in reducing the rate of preterm birth before 26 weeks of gestation. These findings are consistent with the findings of other previous studies, although those did not include cases of RC, but of primary cerclage. Some studies have reported that the postoperative cervical length after a primary cerclage is an important factor in predicting preterm birth (11–14). Phils et al. described that a postoperative CL <20 mm was a predictive factor for early preterm delivery (14). However, these previous studies only included ultrasound-indicated or history-indicated cerclages, excluding cerclage indicated by physical examination or RCs.

In our study, we also analyzed various factors to predict preterm birth and observed that the number of knots did not influence the outcome of RCs. Several previous studies have been conducted that have analyzed this factor. Woensdregt et al. reported no measurable benefit from the placement of double knots over single knots during primary history- and ultrasound-indicated cerclages (15). Furthermore, Maria et al. described that the use of two stitches during history- and ultrasound-indicated cerclage did not improve pregnancy outcomes compared to the use of a single stitch (16). However, Xu et al. (17) demonstrated that double knots in emergency cerclage were more effective in improving pregnancy prolongation and neonatal prognosis. Notably, these studies included cases of primary cerclage performed in a single center or multiple centers. In our study, we included cases of RC with bulging membranes; among the 35 patients, 14 underwent primary cerclages at various other hospitals, all of which utilized double knots. This heterogeneity may have contributed to the differences observed in our study compared with other studies.

According to our study, RC is a relatively safe procedure for women with bulging membranes following a prior cerclage in selected cases. In our study, we observed only one case of chorioamnionitis after RC and no case of intraoperative rupture of membranes, pregnancy loss, or cervical laceration during the surgery. In the case of chorioamnionitis, the patient underwent PEIC at a GA of 16 weeks, followed by RC at a GA of 18 weeks due to a 3-cm sized bulging membrane, and delivery at a GA of 18 + 5 weeks. We also observed that the postoperative CL <20 mm group experienced more premature rupture of membranes and chorioamnionitis than those experienced by the postoperative CL ≥ 20 mm group, and all cases with premature rupture of membranes and chorioamnionitis had a postoperative CL <20 mm. This finding supports the hypothesis that cerclage sutures may act as mechanical barriers, which means preventing direct contact of the membranes with the vaginal flora, to reduce the risk of preterm birth by restoring cervical anatomy.

To the best of our knowledge, our study is the first single-center study to analyze the predictive factors that prevent preterm birth through multiple statistical evaluations and to determine the importance of postoperative CL following RC as a predictor of successful outcomes. However, this study had several limitations. This was a retrospective study with a relatively small sample size. Therefore, selection bias and type II errors were possible. All cases were performed by two skilled and experienced surgeons, which may have influenced our favorable outcomes. Additionally, we used a simple model to evaluate the predictive factors for preterm birth. In future studies, it may be beneficial to use different methodological validations for preterm birth risk estimation (18). To overcome these limitations, a multicenter, randomized trial is needed to assess the effectiveness of RC. Additionally, prospective studies on biomarkers in amniotic fluid, serum, and placenta are recommended to determine the underlying mechanisms of RC and other predictive values to improve obstetric outcomes.

## Conclusion

Our study may provide promising results regarding the effectiveness and safety of RC after the failure of prior cerclage in selected cases. Furthermore, a postoperative CL ≥20 mm following repeat cerclage could be a predictive factor to prevent preterm birth before 26 weeks of gestation.

## Data availability statement

The raw data supporting the conclusions of this article will be made available by the authors, without undue reservation.

## Author contributions

SP designed and performed statistics, analyzed data, and wrote the manuscript. K-YL provided the data. J-ES provided the data, designed and supervised the manuscript, and approved the final version for publication. All authors contributed to the article and approved the submitted version.
