# Supplementary material for: Postoperative cervical length to predict success of repeat cerclage in singleton pregnancies with prolapsed membranes after prior cerclage
Source: Front Med (Lausanne). 2023 Aug 21;10:1248321. doi: 10.3389/fmed.2023.1248321 (PMC10475578; doi:10.3389/fmed.2023.1248321)
Supplement: Supplementary file 1 [file Table_1.docx]

**Supplementary table 1. Comparison of characteristics and outcomes according to types of prior cerclage**

|  | History-indicated cerclage  (n=20) | Ultrasound-indicated cerclage  (n=5) | Physical examination-indicated cerclage  (n=10) | *p*-value |
| --- | --- | --- | --- | --- |
| Maternal age | 34 [29─42] | 33 [29─35] | 35 [30─40] | 0.49 |
| BMI | 25.2 [20.4─30.8] | 26.5 [21.8─38.8] | 25.4 [19.5─27.6] | 0.24 |
| Parity |  |  |  | 0.86 |
| Primiparous | 8/20 (40%) | 2/5 (40%) | 3/10 (30%) |  |
| Multiparous | 12/20 (60%) | 3/5 (60%) | 7/10 (70%) |  |
| History of PTB | 20/20 (100%) | 3/5 (60%) | 4/10 (40%) | 0.001* |
| History of conization/LEEP | 0/20 (0%) | 1/5 (20%) | 0/10 (0%) | 0.01* |
| Mullerian anomaly | 1/20 (5%) | 1/5 (20%) | 0/10 (0%) | 0.28 |
| In vitro fertilization | 5/20 (25%) | 0/5 (0%) | 3/10 (30%) | 0.40 |
| GA at prior cerclage (weeks) | 13+3 [12+5─15+4] | 17+3 [15+3─20+1] | 17+6 [13+2─20] | <0.001* |
| GA at repeat cerclage (weeks) | 21+4 [16+2─23+6] | 21 [19─22+5] | 21 [17+2─23+4] | 0.95 |
| Latency between RC and prior cerclage (days) | 56 [20─76] | 20 [17─38] | 20 [5─62] | 0.001* |
| Size of bulging membranes (cm) | 2.0 [1.0─6.0] | 3.0 [1.4─6.0] | 3.0 [1.0─5.0] | 0.32 |
| Culture positive | 10/20 (50%) | 4/5 (80%) | 5/9 (55.6%) | 0.48 |
| NLR before RC | 4.8 [3.0─18.0] | 4.1 [3.3─6.6] | 5.2 [2.8─12.5] | 0.65 |
| CRP before RC | 6.1 [2.6─44.0] | 5.2 [1.4─5.8] | 3.8 [0.9─15.5] | 0.28 |
| NLR after RC | 6.8 [1.9─69.5] | 5.2 [2.9─11.5] | 8.0 [3.7─11.5] | 0.54 |
| NLR after RC | 18.5 [5.1─66.4] | 12.7 [1.8─19.6] | 10.3 [3.6─23.6] | 0.46 |
| Numbers of suture knots |  |  |  | 0.73 |
| Single knot | 11/20 (5.5%) | 3/5 (60%) | 7/10 (70%) |  |
| Double knots | 9/20 (4.5%) | 2/5 (40%) | 3/10 (30%) |  |
| Postoperative CL (mm) | 20.0 [4.6─40.7] | 10.2 [7.7─28.8] | 15.5 [8.0─29.4] | 0.22 |
| GA at delivery (weeks) | 32+2 [18+4─40+3] | 23+6 [20+3─36+3] | 25 [20+5─34+3] | 0.09 |
| Prolongation date (days) | 65 [2─144] | 10 [3─97] | 25 [3─103] | 0.08 |
| Neonatal birth weight (g) | 1845 [160─3710] | 710 [370─2620] | 665 [400─2860] | 0.08 |
| Very low birth weight | 9/20 (45%) | 4/5 (80%) | 8/10 (80%) | 0.11 |
| Extremely low birth weight | 6/20 (30%) | 4/5 (80%) | 7/10 (70%) | 0.04* |
| Viability | 16/20 (80%) | 4/5 (80%) | 7/10 (70%) | 0.82 |
| NICU admission | 11/16 (68.8%) | 3/4 (75%) | 7/7 (100%) | 0.25 |
| Apgar score <7 at 5 minutes | 10/16 (62.5%) | 2/4 (50%) | 2/7 (28.6%) | 0.32 |
| Perinatal death (<72 hr) | 2/16 (12.5%) | 2/4 (50%) | 0/7 (0%) | 0.07 |
| Early neonatal death (<7 days) | 2/16 (12.5%) | 2/4 (50%) | 1/7 (14.3%) | 0.21 |
| Data are expressed as medians (ranges), and numbers (percentages).  **P* <0.05, which means statistical difference.  BMI, body mass index; PTB, preterm birth; LEEP, loop electrosurgical excision procedure; GA, gestational age; RC, repeat cerclage; NLR, neutrophil lymphocyte ratio; CL, cervical length; NICU, neonatal intensive care unit; CRP, c-reactive protein. | | | | |
