# Supplementary material for: Postoperative cervical length to predict success of repeat cerclage in singleton pregnancies with prolapsed membranes after prior cerclage
Source: Front Med (Lausanne). 2023 Aug 21;10:1248321. doi: 10.3389/fmed.2023.1248321 (PMC10475578; doi:10.3389/fmed.2023.1248321)
Supplement: Supplementary file 2 [file Table_2.docx]

**Supplementary table 2. Comparison of characteristics and outcomes according to number of knots**

|  | Single knot (n=21) | Double knots (n=14) | *p-value* |
| --- | --- | --- | --- |
| Maternal age | 33 [29─40] | 36 [31─42] | 0.02 |
| BMI | 25.2 [19.5─38.0] | 25.3 [21.8─28.6] | 0.88 |
| Parity |  |  | 0.72 |
| Primiparous | 7/21 (33.3%) | 6/14 (42.9%) |  |
| Multiparous | 14/21 (66.7%) | 8/14 (57.1%) |  |
| History of PTB | 16/21 (76.2%) | 11/14 (78.6%) | 1.00 |
| History of  conization/LEEP | 0/21 (0%) | 1/14 (7.1%) | 0.40 |
| Mullerian anomaly | 2/21 (9.5%) | 0/14 (0%) | 0.51 |
| In vitro fertilization | 4/21 (19.0%) | 4/14 (28.6%) | 0.69 |
| Prior cerclage type |  |  | 0.73 |
| History-indicated cerclage | 11/21 (52.4%) | 9/14 (64.3%) |  |
| Ultrasound-indicated cerclage | 3/21 (14.3%) | 2/14 (14.3%) |  |
| Physical examination-indicated cerclage | 7/21 (33.3%) | 3/14 (21.4%) |  |
| GA at prior cerclage (weeks) | 13+4 [12+5─20] | 14+4 [13─20+1] | 0.59 |
| GA at repeat cerclage (weeks) | 21+2 [17+2─23+6] | 21+3 [16+2─23+4] | 0.84 |
| Latency between RC and prior cerclage (days) | 41 [8─76] | 33 [5─66] | 0.57 |
| Size of bulging membranes (cm) | 2.8 [1.0─6.0] | 2.0 [1.0─4.0] | 0.10 |
| Culture positive | 8/21 (38.1%) | 11/14 (41.2%) | 0.08 |
| NLR before RC | 4.7 [2.8─8.3] | 5.2 [3.0─18.0] | 0.15 |
| CRP before RC | 5.9 [1.4─42.3] | 5.5 [0.9─44.0] | 0.92 |
| NLR after RC | 5.7 [1.9─12.5] | 7.7 [2.3─69.5] | 0.07 |
| CRP after RC | 14.7 [1.8─23.6] | 16.8 [3.6─66.4] | 0.56 |
| Postoperative CL (mm) | 15 [8─40] | 20 [5─41] | 0.08 |
| GA at delivery (weeks) | 25+2 [20+3─39+5] | 32+5 [18+4─40+3] | 0.06 |
| Prolongation date (days) | 25 [2─144] | 79 [7─139] | 0.05 |
| Neonatal birth weight (g) | 830 [370─3710] | 1980 [160─3030] | 0.20 |
| Very low birth weight | 15/21 (71.4%) | 6/14 (42.9%) | 0.16 |
| Extremely low birth weight | 13/21 (61.9%) | 4/14 (28.6%) | 0.09 |
| Viability | 15/21 (71.4%) | 12/14 (85.7%) | 0.43 |
| NICU admission | 13/15 (86.7%) | 8/12 (66.7%) | 0.36 |
| Apgar score <7 at 5 minutes | 8/15 (53.3%) | 5/12 (41.7%) | 0.70 |
| Perinatal death (<72 hr) | 3/15 (20%) | 1/12 (8.3%) | 0.61 |
| Early neonatal death (<7 days) | 4/15 (26.7%) | 1/12 (8.3%) | 0.34 |
| Data are expressed as medians (ranges), and numbers (percentages).  **P* <0.05, which means statistical difference.  BMI, body mass index; PTB, preterm birth; LEEP, loop electrosurgical excision procedure; GA, gestational age; RC, repeat cerclage; NLR, neutrophil lymphocyte ratio; CL, cervical length; NICU, neonatal intensive care unit; CRP, c-reactive protein. | | | |
